# Fixed low dose versus concentration-controlled initial tacrolimus dosing with reduced target levels in the course after kidney transplantation: results from a prospective randomized controlled non-inferiority trial (Slow & Low study)

**DOI:** 10.1016/j.eclinm.2023.102381

**Published:** 2023-12-22

**Authors:** Julian Stumpf, Klemens Budde, Oliver Witzke, Claudia Sommerer, Thomas Vogel, Peter Schenker, Rainer Peter Woitas, Mirian Opgenoorth, Evelyn Trips, Eva Schrezenmeier, Christian Hugo, German S&L Study, Matthias Girndt, Gunter Wolf, Christine Kurschat, Kai Lopau, Jens Lutz

**Affiliations:** a Division of Nephrology, Department of Internal Medicine III, University Hospital Carl Gustav Carus at the Technische Universität Dresden, Dresden, Germany; b Department of Nephrology and Medical Intensive Care, Charité Universitätsmedizin Berlin, Berlin, Germany; c Department of Infectious Diseases, West German Centre of Infectious Diseases, University Hospital Essen, University Duisburg-Essen, Germany; d Department of Nephrology, Kidney Center, University Hospital Heidelberg, Heidelberg, Germany; e Department of General, Visceral and Transplant Surgery, University Hospital Münster, Münster, Germany; f Department of Surgery, University Hospital Knappschaftskrankenhaus Bochum, Ruhr-University Bochum, Bochum, Germany; g Department of Internal Medicine I, University of Bonn, Bonn, Germany; h Department of Nephrology and Hypertension, University Hospital of Erlangen, Erlangen, Germany; i Coordination Centre for Clinical Trials, Faculty of Medicine Carl Gustav Carus, Dresden University of Technology, Dresden, Germany; j Berlin Institute of Health at Charité-Universitätsmedizin Berlin, BIH Academy, Clinician Scientist Program Universitätsmedizin Berlin, Berlin, Germany

**Keywords:** Renal transplantation, Tacrolimus monitoring, Fixed low dose, Immunosuppression

## Abstract

**Background:**

Optimal initial tacrolimus dosing and early exposure of tacrolimus after renal transplantation is not well studied.

**Methods:**

In this open-label, 6 months, multicenter, randomized controlled, non-inferiority study, we randomly assigned 432 renal allograft recipients to receive basiliximab induction, mycophenolate and steroids and either standard prolonged-release tacrolimus (trough levels: 7–9 ng/ml; Standard Care arm), or an initial 7-day fixed 5 mg/day dose of prolonged-release tacrolimus followed by lower tacrolimus predose levels (trough levels: 5–7 ng/ml; Slow & Low arm). The primary end point was the combined incidence rate of biopsy-proven acute rejections (BPAR; including borderline), graft failure, or death at 6 months with a non-inferiority margin of 12.5%. (EudraCT-Nr: 2013-001770-19.

**Findings:**

The combined primary endpoint in the Slow & Low arm was non-inferior compared to the Standard Care arm (22.1% versus 20.7%; difference: 1.4%, 90% CI −5.5% to 8.3%). The overall rate of BPAR including borderlines was similar (Slow & Low 17.4% versus Standard Care 16.6%). Safety parameters such as delayed graft function, kidney function, donor specific HLA-antibodies, infections, or post-transplantation diabetes mellitus did not differ.

**Interpretation:**

This is the first study to show that an initial fixed dose of 5 mg per day followed by lower tacrolimus exposure is non-inferior compared to standard tacrolimus therapy and equally efficient and safe within 6 months after renal transplantation. These data suggest that therapeutic drug monitoring for prolonged release tacrolimus can be abandoned until start of the second week after transplantation.

**Funding:**

Investigator-initiated trial, financial support by Astellas Pharma GmbH.

**Research in context**

**Evidence before this study**

Based on the ELITE Symphony study published in 2007, immunosuppressive therapy with low-dose tacrolimus, mycophenolic acids and steroids after induction with an interleukin-2 receptor antibody has become the standard of care worldwide after kidney transplantation, but the perfect therapeutic window between safety/toxicity and efficacy of tacrolimus dosing/monitoring and handling has not been well-defined. While drug monitoring is highly recommended for this initial phase, mostly daily trough level monitoring with frequent tacrolimus dose changes as done in many transplant centers add to drug level variability and potentially toxicity. A search in PubMed and Google Scholar Search Engines through August 16, 2023, using the keywords “renal transplantation”, “immunosuppression”, “tacrolimus”, and “fixed dose” did not reveal any prospective studies that examined fixed doses of tacrolimus in the immediate early phase after renal transplantation.

**Added value of this study**

At 6-months after kidney transplantation the Slow & Low, fixed dose tacrolimus dosing arm showed non-inferiority compared to the standard immunosuppressive therapy regarding the combined endpoint of BPAR, graft loss, and death. This overall result was applicable to a wide range of transplant recipients, robust and independent of the inclusion or exclusion of borderline or protocol rejections in the primary endpoint. The low fixed dose concept was extremely easy to handle and safe. This study also demonstrated that early reduced tacrolimus exposure leads to an earlier apparence of BPARs, a slight severity shift of borderline towards BANFF Ia rejections (both post-hoc analysis) without (pre-specified analyzed) changing incidences of delayed graft function, kidney function, post transplantation diabetes mellitus, infections or development of donor specific antibodies.

**Implications of all the available evidence**

This is the first randomized controlled study indicating that therapeutic drug monitoring for tacrolimus can be abandoned until start of the second week after renal transplantation using a fixed dose of 5 mg prolonged released tacrolimus. The overall result of non-inferiority of the Slow & Low fixed dose tacrolimus arm compared to the current standard immunosuppressive regimen demonstrates this novel approach efficient and safe and may be a cost effective alternative therapy due to its simplified handling of patients early after transplantation. On the other hand, lowering early tacrolimus exposure as done in our study, while still demonstrating sensitivity on timing and severity (but not incidence) of BPAR, is not sufficient for improving incidences of delayed graft function, kidney function, post transplantation diabetes mellitus, or infections after renal transplantation.

## Introduction

Based on the findings of the ELITE-Symphony study, the current KDIGO guidelines recommend as standard of immunosuppressive therapy after renal transplantation an induction therapy with tacrolimus, mycophenolate (MPA), and steroids.^1^^,^^2^ However, several studies of large data sets including the Symphony trial linked currently used tacrolimus blood trough levels to nephrotoxic effects affecting kidney function as well as incidence of delayed graft function (DGF) (especially in marginal donor organs).^3^^,^^4^ These data suggest that even lower tacrolimus trough levels should be aimed for, which however might lead to higher rejection rates, which also could have a negative impact on kidney function.^4^ A large post-hoc study investigating >1300 renal allograft recipients showed no significant correlation of tacrolimus trough levels with the incidence of biopsy proven acute rejections (BPAR).^5^ Tacrolimus predose concentrations at five time points posttransplant (days 3, 10, 14, months 1 and 6 after transplantation) were not associated with the incidence of acute rejections, neither in the first month nor within the rest of the first year.

While these data clearly support further investigations on the optimal initial tacrolimus dosing and levels in the early phase after transplantation, many transplant centers use daily tacrolimus trough level determinations aiming at a therapeutic window, which however so far has not been well-defined.^5^ Despite the basic pharmacokinetic principles that tacrolimus saturation normally takes four days to achieve a stable equilibrium, most transplant physicians tend to measure trough levels not at steady state but rather correct drug levels by frequent dose changes (some on a daily basis). In combination with fear of rejection due to low levels, this might lead to overdosing with high drug levels and an increased risk for side effects such as nephro- or neurotoxicity, DGF, or posttransplantation diabetes mellitus (PTDM). In addition, high corticosteroid doses causing Cyp450 enzyme induction and variable absorption during the first week after transplantation may further amplify drug level variability.^6^ After the first week, corticosteroid doses are down to 20 mg/day and bowel movement is usually well-established leading to a more stable tacrolimus absorption and metabolism.^7^ Thus, tacrolimus dose changes starting at week two may be better correlated with trough levels and result in less overexposure and toxicity.

Based on the set of problems described above, we had the hypothesis that early low and fixed tacrolimus dosing may be a safe and practical alternative. Furthermore, the lack of a relationship between tacrolimus trough levels and the incidence of BPAR prompted us to initiate an open-label, randomized, multicenter study comparing the standard approach to an initial fixed 5 mg dose of prolonged release tacrolimus administered once daily for the first six days after transplantation without trough level determinations and reduced tacrolimus trough levels thereafter. The primary objective was non-inferiority of a combined endpoint of the incidence of BPAR (including borderlines), graft loss, and death at six months after transplantation.

## Methods

### Study design

We conducted an investigator-initiated, prospective, randomized, open-label, multicenter study with two parallel study arms of adult renal transplant recipients in fourteen German centers (EudraCT-Nr: 2013-001770-19). The study was carried out in compliance with the provisions of the Declaration of Helsinki and Good Clinical Practice guidelines. All patients provided written informed consent and were allowed to withdraw from the study at any time. The safety population and the intention-to-treat population consisted of all patients who received at least one dose of a study drug and underwent successful renal transplantation. The per-protocol population was defined, including only patients who received treatment without severe protocol violations (see Supplementary Methods) throughout the study period.

Demographic and baseline data of the recipients and donors were assessed before transplantation including sero-status for cytomegalovirus (CMV) and Epstein Barr virus (EBV). Documentation of clinical signs and laboratory data were obtained at baseline, day 6, and at months 1, 3, and 6.

Important to note that external monitoring was performed throughout the study period. An independent data safety monitoring board consisting of one statistician and two international transplant physicians evaluated overall study safety after inclusion of 40, 80, and 200 patients.

### Randomization

Participants were centrally randomly assigned via a web-based randomization algorithm in a 1:1 ratio after signing informed consent before transplantation. The allocation list was computer-generated by block randomization with blocks of length four using nQuery-Advisor® 6.01 and stratified by trial site, living donation, and transplantation via European Senior Program (ESP). The randomization algorithm was incorporated in the electronic data management system and after obtaining informed consent the trial sites were allowed to request a patient's randomization result.

### Study medication

Patients were randomized to one of two study arms (Standard Care and Slow & Low) as indicated by Supplementary Figure S1.

All patients received induction therapy with basiliximab (Simulect®, Novartis, 20 mg intravenously on day 0 before allograft reperfusion and day 4), prolonged release tacrolimus administered once daily (Advagraf®, Astellas Pharma GmbH), 2 × 1 g/die mycophenolate mofetil (MMF) (CellCept®, Roche Pharma AG), or enteric coated mycophenolic sodium (EC-MPS) at equivalent doses, and prednisolone (Solu Decortin®, Merck Serono), all of which were started before transplantation. Prednisolone/Methylprednisolone was given at center standard, but tapering had to be done to reach 20 mg/16 mg after four weeks, 10 mg/8 mg after eight weeks, and 5 mg/4 mg daily after 12 weeks, respectively.

In the Standard Care arm, tacrolimus starting dose was 0.2 mg/kg once daily (as recommended in the product information) followed by tacrolimus trough levels of 7–9 ng/ml in the first two months and 6–8 ng/ml from month 3 to 6. Trough levels until day 6 had to be determined on a daily basis and later according to center standard.

In the Slow & Low arm, prolonged release tacrolimus was started once daily at a fixed dose of 5 mg before and for the first six days after transplantation. Trough levels until day 6 had to be determined on a daily basis but were blinded to the investigators. The first unblinded tacrolimus trough level on day 6 led to the first dose correction on day 7 by the investigators. From day 7 on, tacrolimus trough levels were adapted to reduced levels of 5–7 ng/ml in the first two months and 4–6 ng/ml from month 3–6. For the event of reaching a potentially toxic tacrolimus drug level of 20 μg/ml or higher on two days, a specific alarm system was implemented to unblind and inform the investigators for correction of the fixed 5 mg dose of tacrolimus.

The aim of the fixed dose of 5 mg prolonged release tacrolimus was to establish a bottom up principle to avoid excessive trough levels in the initial phase (see also introduction). According to the data of Crespo et al., the prolonged release tacrolimus level/dose factor in the first week after renal transplantation is 81, which at a trough level around 6 ng/ml would correspond to an initial dose of 0.07 mg/kg body weight.^7^ For weight ranges of 50–100 kg, this would correspond to starting doses of 4–7 mg prolonged release tacrolimus daily. However, since weight-adjusted doses often lead to significant trough level outliers, especially at very low or very high body weights, a fixed starting dose of 5 mg was specified to the mean weight range of 70 kg. In addition, 5 mg is the equivalent of a single capsule also simplifying dosage and daily tacrolimus management.

### Inclusion and exclusion criteria

Only adult candidates ≥18 years with low immunological risk who were scheduled to receive a single AB0-compatible organ renal transplant from either a living or deceased donor were eligible with a negative complement-dependent cytotoxicity cross-match. Patients receiving a second renal transplant were eligible, if first allograft was not lost due to acute rejection within the first year after transplantation. Patients with pre-transplant existing donor-specific HLA antibodies were not eligible and recipients had to have a current PRA level ≤20%. Further exclusion criteria are reported in Supplementary Methods.

### Efficacy end points

The primary efficacy outcome was the combined end point of the incidence of BPAR including borderline rejection, graft failure, and death within the first 6 months after renal transplantation. All suspected episodes of acute rejection were confirmed by biopsy, with histologic characteristics described according to the Banff criteria of 2013.^8^ Protocol biopsies were allowed but not mandatory. In sensitivity analyses, the occurrence of borderline rejection and detection of rejection by protocol biopsy were not counted as event in the primary endpoint.

### Secondary end points

Secondary end points were renal function (serum creatinine and calculated GFR by the MDRD-IV, Nankivell or CKD-EPI equation), rate of delayed graft function (DGF), rate of chronic active antibody-mediated rejections, rate of donor-specific human leukocyte antigen antibodies at 6 months, number of tacrolimus dose modifications, incidence of PTDM, infections including the incidence of CMV (PCR >400 copies/μl), EBV (PCR >400 copies/μl), or BKV (PCR >1000 copies/μl), incidence of BKV nephropathy, and incidence of malignancy.^9^ For PTDM assessment (according to the ADA recommendations), fasting glucose level was determined at each visit (day 6, months 1, 3 and 6); HbA1c level was measured at each visit except at day 6, and an oral glucose tolerance test (OGTT) was performed at months 1 and 6 in all patients without a current diagnosis of PTDM (considering diagnosis before and after transplantation). Additionally, the occurrence of prediabetes (according to ADA recommendations) was assessed (post-hoc). Additional sensitivity analyses of PTDM and prediabetes were done excluding transient post-transplant events in the early post-transplant period reported i. e. from day 1 to day 27 or day 59.

### Sample size calculation

The projected incidence of our combined primary end point BPAR/graft loss/death within 6 months (P_A_) was estimated at 20% for our reference Standard Care arm.^1^^,^^10^ According to our working hypothesis that an alternative tacrolimus dosing with lower tacrolimus exposure is not accompanied by an increased BPAR/graft loss/death rate in this immunologically low risk population compared to standard tacrolimus treatment, we assumed the same 20% failure rate (P_B_) for the Slow & Low arm.^11^^,^^12^ The aim of our study was to show non-inferiority of the Slow & Low arm versus the Standard Care arm with respect to the primary endpoint with a non-inferiority margin of 12.5 percent.

The sample size was designed for the null hypothesis P_B_—P_A_ ≥12.5 percent to show non-inferiority of the Slow & Low versus the Standard Care arm with a statistical power of 90% and a significance level of 0.05. We calculated that 360 patients should be allocated to two study groups of 180 patients each. To compensate a drop-out rate of 10%, we aimed to recruit a total of 400 patients (nQuery-Advisor® 6.01).

### Statistical analysis

All analyses were performed on the basis of an intention-to-treat principle. Categorical variables were summarized as counts and percentages, and continuous variables as means with standard deviations or median and interquartile range as appropriate. Primary endpoint was tested by one-sided test of equivalence with a non-inferiority margin of 12.5% and a significance level of 5 percent. Accordingly, two-sided 90 percent confidence interval is presented for the absolute risk difference in primary endpoint. Categorical data were compared using the Fisher's exact test or Chi-square test, and continuous variables using the Wilcoxon-Mann-Whitney U-test or t-test. Courses of laboratory values were analyzed by linear mixed models with patient as random factor. The time for reaching BPAR, graft loss, or death was illustrated by the Kaplan-Meier curve and cumulative incidence curve for BPAR alone but no statistical test was performed. Graft survival (counting graft loss and death as event; unplanned complementary analysis) and time to PTDM were analyzed by Kaplan-Meier method and tested by log-rank test. Treatment effect was estimated by absolute risk difference for categorical data and hazard ratio for time-to-event data along with 95% two-sided confidence interval. Subgroup analyses were planned for strata of randomization (living and postmortem donation, patients with and without transplantation via ESP) and donors with expanded criteria.^13^ No non-inferiority margin was defined for subgroups or sensitivity analyses. A per-protocol analysis was prespecified, see Supplementary Material for reasons for exclusion. Analyses were performed using SAS software 9.4. Sensitivity, subgroup, and per-protocol analyses of the primary endpoint were presented by Forest plot (Review Manager Version 5.4). All secondary and explorative analyses were performed in a descriptive manner. A follow-up for data collection was done whenever possible with patients who terminated prematurely study treatment. Missing endpoint data was not imputed. The study design did not include an interim analysis of efficacy.

### Role of the funding source

The trial was designed and run by the last author, who received financial support from Astellas Pharma GmbH, Germany. The funder had no role in data collection, data analysis, data interpretation, or writing of the manuscript.

## Results

### Patient population

**Fig. 1:**
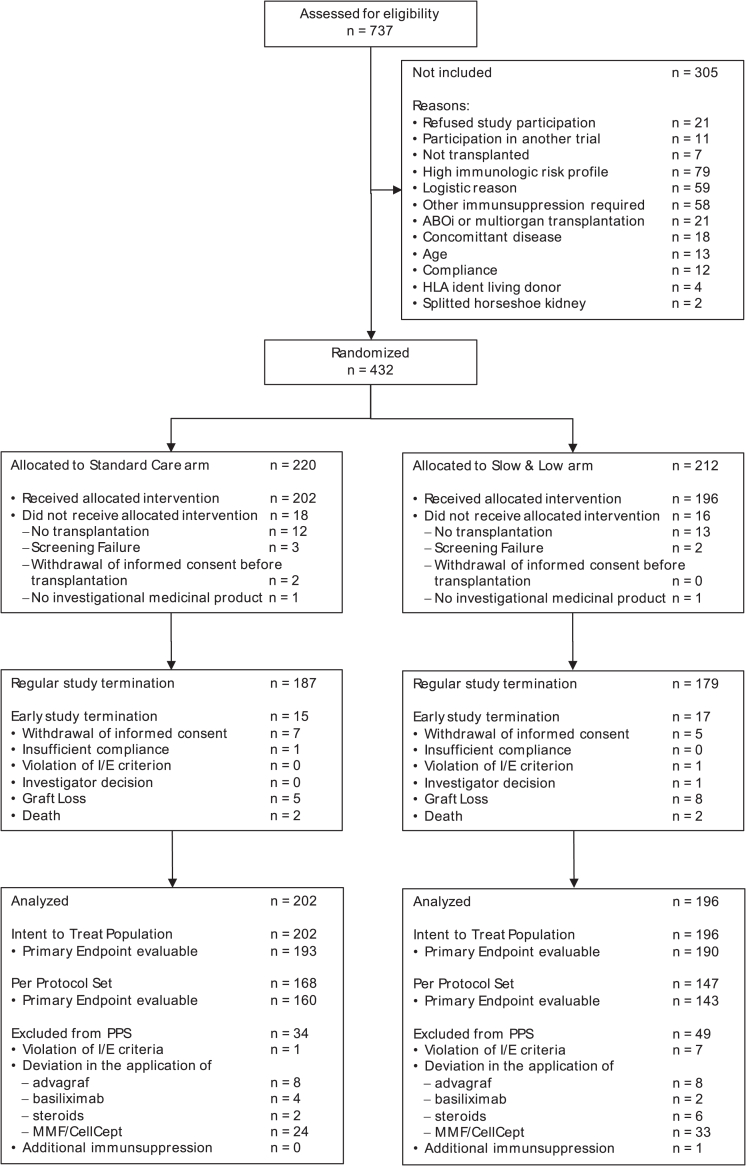
Study flow chart. The per-protocol population comprises all patients who received the allocated treatment without severe protocol violation during the complete study follow up period of 6 months (see Supplemetary Material). Multiple severe protocol violations may have occurred in one patient. PPS = per protocol set.

Fourteen German transplantation centers enrolled and randomized 432 patients between 15th of November 2014 and 17th of July 2018, of which 398 received allocated treatment. Of these 398 patients, 202 patients received treatment in the Standard Care control arm, 196 patients in the Slow & Low arm (Fig. 1). 92% of all study patients reached the regular end of study at 6 months, without differences between arms (Fig. 1). Only 3.0% (12/398) of all study participants withdrew informed consent after renal transplantation before regular end of study, one patient was excluded due to incompliance, one by investigator decision, one by violation of exclusion/inclusion criteria, and 4.3% (17/398) underwent either graft loss or death (Fig. 1).

Baseline characteristics of the study arms were well balanced and did not differ among both groups (Table 1). For example, panel-reactive antibodies were found in 15 patients only. Six of them were randomized to the Control arm and nine of them to the Slow & Low arm (Table 1).

**Table 1:** Baseline characteristics.

	Standard care arm	Slow & Low arm	Total
	N = 202	N = 196	N = 398
Recipient age [years]	54 ± 13	55 ± 12	55 ± 13
Recipient age ≥65 years [(n (%)]	55 (27.2%)	50 (25.5%)	105 (26.4%)
Male sex [n (%)]	131 (64.9%)	128 (65.3%)	259 (65.1%)
Body mass index [kg/m^2^]	26.5 ± 4.2	27.2 ± 4.8	26.8 ± 4.5
Cause of end-stage renal disease			
Glomerulonephritis [n (%)]	61 (30.2%)	68 (34.7%)	129 (32.4%)
Diabetes mellitus [n (%)]	17 (8.4%)	14 (7.1%)	31 (7.8%)
Arterial hypertension [n (%)]	17 (8.4%)	16 (8.2%)	33 (8.3%)
Pyelonephritis or Interstitial nephritis [n (%)]	10 (5.0%)	8 (4.1%)	18 (4.5%)
Cystic or polycystic kidney disease [n (%)]	38 (18.8%)	25 (12.8%)	63 (15.8%)
Systemic autoimmun diseases^a^ [n (%)]	5 (2.5%)	6 (3.0%)	11 (2.8%)
Reflux nephropathy [n (%)]	6 (3.0%)	4 (2.0%)	10 (2.5%)
Other^b^ [n (%)]	48 (23.8%)	55 (28.1%)	103 (25.9%)
Medical history			
Diabetes mellitus [n (%)]	34 (16.8%)	30 (15.3%)	64 (16.1%)
Arterial hypertension [n (%)]	192 (95.0%)	190 (96.9%)	382 (96.0%)
Coronary heart disease [n (%)]	51 (25.2%)	47 (24.0%)	98 (24.6%)
Cerebrovascular disease [n (%)]	6 (3.0%)	6 (3.1%)	12 (3.0%)
Peripheral arterial disease [n (%)]	19 (9.4%)	15 (7.7%)	34 (8.5%)
Heart Failure [n (%)]	38 (18.8%)	18 (9.2%)	56 (14.1%)
Asthma or COPD [n (%)]	10 (5.0%)	16 (8.2%)	26 (6.5%)
Malignancy [n (%)]	8 (4.0%)	22 (11.2%)	30 (7.5%)
Dialysis waiting time of recipients [months]	73.3 ± 43.9	71.8 ± 45.5	72.6 ± 44.6
Type of donor			
Deceased [n (%)]	167 (82.7%)	163 (83.2%)	330 (82.9%)
Living [n (%)]	35 (17.3%)	33 (16.8%)	68 (17.1%)
Donor with expanded criteria [n (%)]	105 (52.0%)	107 (54.6%)	212 (53.3%)
Donor age (years)	56 ± 14	57 ± 12	56 ± 13
Donor age ≥65 years [n (%)]	56 (27.7%)	52 (26.5%)	108 (27.1%)
Antigen mismatches: A, B, and DR (no)	3.0 ± 1.7	2.9 ± 1.7	3.0 ± 1.7
No panel-reactive antibodies before transplantation [n (%)]	196 (97.0%)	187 (95.4%)	383 (96.2%)
Previous transplants [n (%)]	11 (5.4%)	4 (2.0%)	15 (3.8%)
Cold-ischaemia time of deceased donor grafts (min)	703.4 ± 230.5	719.3 ± 243.1	711.3 ± 236.6
Cytomegalovirus serologic status			
Missing [n (%)]	8 (4.0%)	6 (3.1%)	14 (3.5%)
donor negative, recipient negative (low risk) [n (%)]	31 (15.3%)	43 (21.9%)	74 (18.6%)
donor positive/negative, recipient positive (intermediate risk) [n (%)]	99 (49.0%)	92 (46.9%)	191 (48.0%)
donor positive, recipient negative (high risk) [n (%)]	64 (31.7%)	55 (28.1%)	119 (29.9%)
Epstein–Barr virus serological status			
Missing [n (%)]	23 (11.4%)	16 (8.2%)	39 (9.8%)
donor negative, recipient negative (low risk) [n (%)]	1 (0.5%)	1 (0.5%)	2 (0.5%)
donor positive/negative, recipient positive (intermediate risk) [n (%)]	170 (84.2%)	173 (88.3%)	343 (86.2%)
donor positive, recipient negative (high risk) [n (%)]	8 (4.0%)	6 (3.1%)	14 (3.5%)

Data are presented as mean ± standard deviation, or absolute and relative frequencies.

a Comprises vasculitis, systemic lupus erythematosus, and hemolytic uremic syndrome.

b Comprises drug induced nephropathy, unkown etiology and other reasons.

**Fig. 2:**
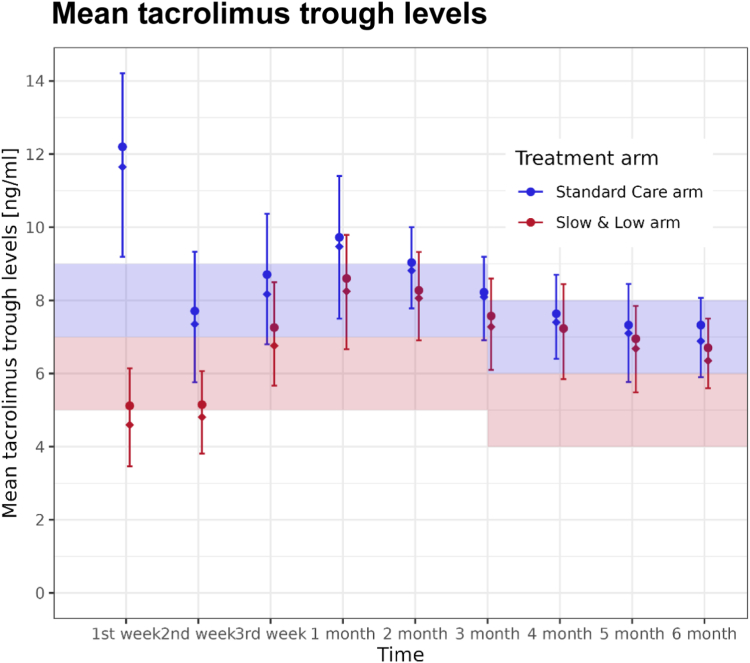
Mean tacrolimus levels during different time periods after renal transplantation. In case, more than one measurement per patient was documented, mean tacrolimus trough level per period was calculated. The groups shown differ in the immunosuppressive regimen which consisted of basiliximab induction, mycophenolate and steroids, and either standard extended-release tacrolimus (according to the dosing recommendation by the manufacturer; Standard Care arm) or a 7-day fixed initial dose of 5 mg/day extended-release tacrolimus followed by lower tacrolimus predose levels (trough levels: 5–7 ng/ml; Slow & Low arm). The colored highlighted areas show the target ranges of the corresponding treatment arms over time. Filled diamond = median, filled circle = mean, strokes = interquartile range (Q1/Q3), if several tacrolimus through values were available between the former and illustrated time point, individual mean values were calculated and used for the graph.

Target trough levels for tacrolimus were within the predefined therapeutic window in the Standard Care arm but only during the first month in the Slow & Low arm (see Fig. 2), thereafter tacrolimus trough levels became more overlapping with the Standard Care arm. For more details also see Supplementary Results (“Induction and maintenance therapy”).

### Efficacy end points

At 6 months, the incidence of the combined primary end point BPAR (including borderline), graft loss, and death was evaluable in 383 of 398 patients and was similar among both study arms (Slow & Low 22.1% (42/190), Standard Care 20.7% (40/193)) meeting the non-inferiority criteria with a difference of 1.4% and a two-sided 90 percent confidence interval from −5.5% to 8.3% (Table 2). The p-value of the one-sided test of equivalence with a non-inferiority margin of 12.5 percent demonstrated non-inferiority (p = 0.004). In the per-protocol analysis, we observed a similar result for the primary endpoint (Slow & Low arm 20.3% 29/143, Standard Care arm 18.8% 30/160, risk difference and two-sided 90% confidence interval 1.5% (−6.0%; 9.0%; one-sided test of equivalence with a non-inferiority margin of 12.5% p = 0.008). Non-inferiority was reached by the Slow & Low arm according to rates of graft loss/mortality and rejections including borderlines in all biopsies. Non-inferiority of the Slow & Low arm was also independent on the definition of BPAR and was reached when BPAR included or excluded borderline rejection. Non-inferiority was also achieved when rejections observed by protocol biopsies were included or excluded.

**Table 2:** Primary and secondary endpoints.

	Standard care arm	Slow & Low arm	Treatment effect^k^ and 90% or 95% CI	p-value
	N = 202	N = 196		
**Primary Endpoint (Efficacy)**				
BPAR, Graft Loss or Death [n/n_evaluable_ (%)]	40/193 (20.7%)	42/190 (22.1%)	1.4 [−5.5; 8.3]	0.004^a^
***Events in primary Endpoint***^b^***[n (%)]***				
BPAR, thereof	32 (16.6%)	33 (17.4%)		
Antibody-mediated rejection	12 (6.2%)	13 (6.8%)		
Acute antibody-mediated rejection Grade I	2 (1.0%)	8 (4.2%)		
Acute antibody-mediated rejection Grade II	9 (4.7%)	5 (2.6%)		
Acute antibody-mediated rejection Grade III	1 (0.5%)	0 (0.0%)		
Chronic active humoral rejections	2 (1.0%)	0 (0.0%)	−1.0 [−2.5; 0.4]	0.499^e^
Borderline Rejection	22 (11.4%)	12 (6.3%)		
T-cell-mediated rejection	10 (5.2%)	22 (11.6%)	6.4 [0.9; 11.9]	0.027^e^
Acute T-cell-mediated rejection Grade IA	2 (1.0%)	11 (5.8%)		
Acute T-cell-mediated rejection Grade IB	0 (0.0%)	2 (1.1%)		
Acute T-cell-mediated rejection Grade IIA	4 (2.1%)	5 (2.6%)		
Acute T-cell-mediated rejection Grade IIB	3 (1.6%)	1 (0.5%)		
Acute T-cell-mediated rejection Grade III	1 (0.5%)	3 (1.6%)		
Chronic active T-cell mediated rejection	1 (0.5%)	2 (1.1%)		
Graft Loss	5 (2.6%)	7 (3.7%)		
Death	4 (2.1%)	2 (1.1%)		
**Secondary endpoints (Safety)**				
Delayed graft function^c^^,^^d^ (acc. to need for dialysis) [n (%)]	22 (10.9%)	21 (10.8%)	−0.1 [−6.3; 6.0]	1.000^e^
Delayed graft function^c^ (assessed by trial site) [n (%)]	42 (20.9%)	31 (16.0%)	−4.9 [−12.5; 2.7]	0.243^e^
Positivity for Anti-HLA antibodies at month 6				
Anti-HLA antibodies investigated [n (%)]	155 (76.7%)	151 (77.0%)		
de novo, thereof [n (%)]	26 (16.8%)	15 (9.9%)	−6.8 [−14.4; 0.7]	0.094^e^
Not donor specific [n (%)]	21 (13.5%)	6 (4.0%)	−9.6 [−15.8; −3.4]	0.004^e^
donor specific [n (%)]	5 (3.2%)	9 (6.0%)	2.7 [−2.0; 7.4]	0.285^e^
***Cardiovascular risk factors***				
Kidney function				
Creatinine [μmol/l]	147.1 ± 56.7	146.3 ± 51.4		0.807^f^
eGFR MDRD-IV [ml/min/1.73 m^2^]	45.9 ± 20.1	45.2 ± 17.6		0.780^f^
eGFR CKD-EPI [ml/min/1.73 m^2^]	48.8 ± 19.6	48.1 ± 18.6		0.762^f^
eGFR Nankivell [ml/min/1.73 m^2^]	59.2 ± 19.7	59.1 ± 18.1		0.977^f^
Systolic blood pressure [mmHg]	135 ± 17.4	136 ± 17.5		0.302^f^
Diastolic blood pressure [mmHg]	79.2 ± 11.5	80.1 ± 10.2		0.194^f^
Cholesterol [mmol/L]	5.2 ± 1.1	5.3 ± 1.2		0.855^f^
HDL [mmol/L]	1.4 ± 0.5	1.4 ± 0.5		0.383^f^
LDL [mmol/L]	3.1 ± 1.0	3.1 ± 1.0		0.644^f^
Weight [kg]	76.8 ± 16.7	77.4 ± 14.9		0.402^f^
BMI [kg/m^2^]^i^	25.5 ± 4.2	26.1 ± 4.7		0.067^f^
Posttransplantation diabetes mellitus (PTDM)				
PTDM evaluable [n (%)]^j^	167 (82.7%)	164 (83.7%)		
No Prediabetes, no PTDM [n (%)]	30 (18.0%)	34 (20.7%)		0.962^e^
Prediabetes, no PTDM [n (%)]	63 (37.7%)	58 (35.4%)		
PTDM [n (%)]	64 (38.3%)	63 (38.4%)		
Prediabetes reported, PTDM unknown [n (%)]	4 (2.4%)	3 (1.8%)		
Unknown [n (%)]	6 (3.6%)	6 (3.7%)		
**Adverse events, infections, other events**				
Patients with				
Any adverse event [n (%)]	200 (99.0%)	193 (98.5%)	−0.5 [−2.7; 1.7]	0.681^e^
Any serious adverse event [n (%)]	134 (66.3%)	136 (69.4%)	3.1 [−6.1; 12.2]	0.522^e^
Infections [n (%)]	123 (60.9%)	113 (57.7%)	−3.2 [−12.9; 6.4]	0.541^e^
CMV infections [n (%)]	32 (15.8%)	25 (12.8%)	−3.1 [−10.0; 3.8]	0.394^e^
Polyoma/BKV infections^h^ [n (%)]	24 (11.9%)	17 (8.7%)	−3.2 [−9.2; 2.8]	0.325^e^
BKV nephropathy^g^ [n (%)]	3 (1.5%)	4 (2.0%)	0.5 [−2.0; 3.1]	0.720^e^
Malignancy [n (%)]	0 (0.0%)	5 (2.6%)	2.6 [0.3; 4.8]	0.028^e^
Neurotoxicities [n (%)]	24 (11.9%)	15 (7.7%)	−4.2 [−10.0; 1.6]	0.179^e^
Anemia [n (%)]	56 (27.7%)	52 (26.5%)	−1.2 [−9.9; 7.5]	0.822^e^

BPAR = biopsy proven acute rejection, eGFR = estimated glomerular filtration rate, IFTA = interstitial fibrosis and tubular atrophy, BKV = BK virus = human polyomavirus 1, HLA = human leukocyte antigen, BMI = body mass index calculated as kilogramm divided by squared body height in meter, CI = confidence interval.

a Non-inferiority test with non-inferiority margin 12.5%.

b Multiple answers possible.

c One patient in arm A und 2 patients in arm B terminated study before day 7.

d Defined as need for dialysis after transplantation, i. e. at least one dialysis within 4 days after TX followed by at least one other dialysis until day 7. The need for dialysis must not be caused by graft rejection or graft loss.

e Fisher's exact test (two-sided).

f Two-sided tested by mixed model including measurements at day 6, month 1, 2 and 6. Values of 6 months visit reported.

g Proven by biopsy.

h Indicated by a viral blood load of more than 1000 copies/μl.

i Body mass index calculated as weight [kg]/height [m]^2^.

j Excluding patients with preexisting diabetes mellitus (arm A n = 34, arm B = 30) and without data on PTDM due to early study termination until day 6 (arm A n = 1, arm B n = 2). For treatment effect, see Fig. 4A and B.

k Treatment effect calculated as absolute risk difference between Slow & Low arm and Standard Care arm for categorical data. Two-sided 90 percent confidence interval for primary endpoint, otherwise, two-sided 95% confidence interval.

### Secondary analyses

**Fig. 3:**
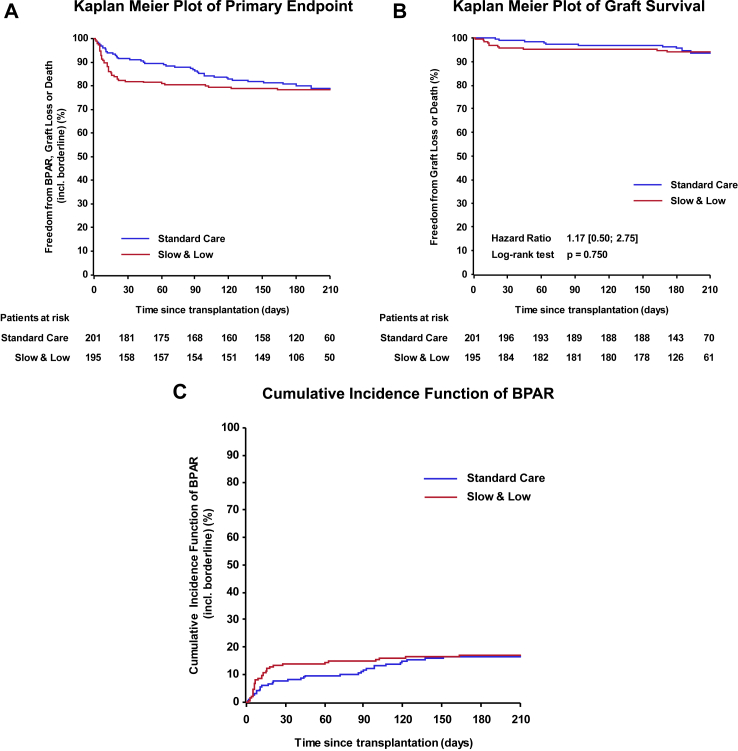
Kaplan-Meier diagram of the primary endpoint (panel A) and allograft survival (panel B) and cumulative incidence function of biopsy-proven acute rejections (panel C) according to treatment arm in the intent to treat population.

Most primary endpoint events (especially BPAR and graft losses) in the Slow & Low arm occurred within the first month after transplantation (35/42–83.3%). In contrast, the events in the Standard Care arm were more equally distributed over the full six months observation period having only 17/40–42.5% events within the first month (Fig. 3A–C, post-hoc analysis). Graft survival after 6 months was excellent and equivalent in both arms with 94.2% (Slow & Low arm) versus 95.7% (Standard Care arm), log rank p-value 0.750 (Fig. 3B). Reasons for the fourteen graft losses were mostly primary non-function (n = 11), of which at least four were due to technical problems. One graft was lost due to a malignant donor lymphoma. Only two graft losses appeared to be due to chronic graft rejection/fibrosis. While overall BPAR rates including borderlines were similar in the Slow & Low arm (33/190–17.4%) versus the Standard Care arm (32/193–16.6%), a statistically significant difference in regard to the severity of BPAR according to BANFF classification was seen in the post-hoc analysis (Table 2). The number of T-cell mediated BPAR graded higher than borderline (BANFF grade IA to III) was increased in transplant recipients treated in the Slow & Low arm (11.6%–22/190) compared to the Standard Care arm (5.2%–10/193; p-value of 0.027). Within the group of T-cell mediated rejections, a certain shift from numerically less borderline rejections in the Slow & Low arm (6.3% (12/190) versus the Standard Care arm 11.4% (22/193) to slightly more severe rejections, in particular BANFF grade IA rejections (Slow & Low arm with 5.8% (11/190) versus Standard Care arm 1.0% (2/193) was noted (Table 2). In contrast, rates of antibody-mediated rejections were similar in both groups (Slow & Low 6.8%–13/190) versus Standard Care 6.2%–12/193).

Despite reaching the margin of non-inferiority in the Slow & Low arm versus Standard Care, the frequency of protocol and clinically indicated biopsies was numerically even slightly higher in the Slow & Low arm: 40 protocol biopsies in 38 patients (19.4%) and 117 clinically indicated biopsies in 67 patients (34.2%), Standard Care arm: 30 protocol biopsies in 30 patients (14.9%) and 107 clinically indicated biopsies in 60 patients (29.7%), exploratory analysed.

### Secondary end points

Development of donor-specific antibodies at six months occurred at a low frequency and was not different in both arms (see Table 2). The *de novo* occurrence of any anti-HLA antibodies at the 6 month time point was investigated in 77 percent of patients in both arms and numerically reduced in the Slow & Low arm (9.9%–15/151) versus the Standard Care arm (16.8%–26/155), p-value 0.094, mainly due to non-donor-specific antibodies (see Table 2).

The frequency of delayed graft function (DGF) was not different in both treatment groups according to two different definitions (Table 2). The DGF rate was 10.8% in the Slow & Low arm and 10.9% in the Standard Care arm (p-value = 0.969), when DGF was defined as need for dialysis after transplantation as described before. DGF definition according to center assessment was also not different in the Slow & Low arm (16.0%–31/194) versus Standard Care arm (20.9%–42/201), p-value = 0.208.

Kidney function as determined by the mean calculated glomerular filtration rate using different formulas (MDRD-IV, CKD-EPI, Nankivell-ratio) was similar at all study time points examined (Table 2).

### Safety end points

#### Adverse events, infections, anemia, and cancer

The two study arms had a similar incidence of adverse (AE) and serious adverse events (SAE) (Table 2). The total number of adverse events for the Slow & Low and Standard Care arm was 1930 and 2157, while the total number of serious adverse events was 327 and 369, respectively.

The overall incidence of all infections, CMV or BKV infections was similar in both arms (Table 2). Biopsy-proven BKV nephropathy was a rare event and occurred at a similar rate in both groups within study period. In the per-protocol population (PP), a tendency towards a reduced incidence of BKV infection and nephropathy was observed. Hereby, the incidence of a BKV infection showed 4.8% (7/147) in the Slow & Low arm versus 10.1% (17/168) in the Standard Care arm (p = 0.089). Biopsy-proven BKV nephropathy was not detected in Slow & Low arm patients (0/147), but in 1.2% (2/168) in Standard Care arm patients (p = 0.501).

Cancer developed in 5 patients during the study period all of them belonging to the Slow & Low arm (Fisher's exact p = 0.028, Table 2). Neurotoxicity was reported in 15/196 patients in the Slow & Low arm (7.7%) compared to 24/202 patients in the Standard Care arm (11.9%, Fisher's exact p = 0.179) and anemia in 52/196 (26.5%) and 56/202 (27.7%) patients in the Slow & Low and Standard Care arm (Fisher's exact p = 0.822), respectively.

#### Metabolic profiles

**Fig. 4:**
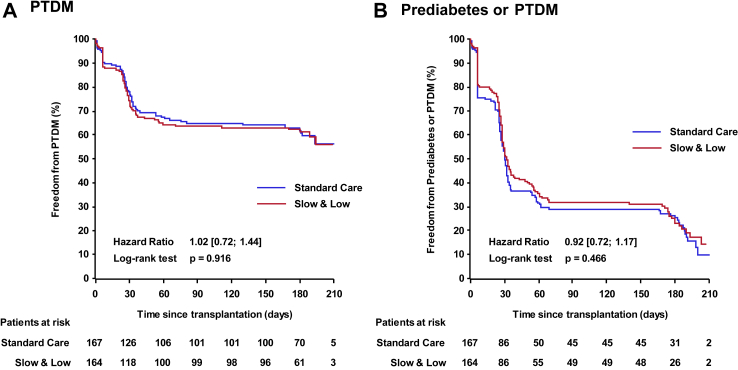
Kaplan-Meier diagram of post transplant diabetes mellitus (PTDM) (panel A) and prediabetes or PTDM (panel B) in the intent-to-treat population according to treatment arm. Patients with preexisting diabetes mellitus were excluded.

Occurrence of PTDM was defined according to the ADA criteria, and evaluations took into consideration the current consensus conference recommendations on PTDM.^14^ Overall incidence of PTDM was similar in both treatment arms, where 63/164 (38.4%) Slow & Low arm recipients without preexisting diabetes mellitus fulfilled at least one ADA criteria during study period and 65/167 (38.9%) in the Standard Care arm (Fig. 4A, Table 2). When inclusion of transient post-transplant hyperglycemia in the early post-transplant periodwas avoided and only the time period from day 28 or day 60 to day 180 was considered, PTDM results were also not different (day 28–180: 55/153 (35.9%) for Slow & Low arm and 59/156 (37.8%) for the Standard Care arm, Fisher's exact p = 0.81; day 60–180: 45/152 (29.6%) in Slow & Low arm and 51/156 (32.7%) in the Standard Care arm, Fisher's exact p = 0.62).^14^

Similarly, occurrence of prediabetes (excluding full diabetes) as defined by ADA criteria (fasting blood glucose level between 5.6 and 6.9 mmol/l, OGTT between 7.8 and 11.0 mmol/l, HbA1c level between 5.7% and 6.4%) was reported in 61/164 patients (37.2%) in the Slow & Low arm and 67/167 patients (40.1%) in the Standard Care arm (Table 2). Prediabetes incidence from day 28–180 was 60/156 (38.5%) for Slow & Low arm and 64/159 (40.3%) for Standard Care arm, p = 0.82; and from day 60–180: 53/151 (35.1%) in Slow & Low arm and 50/155 (32.3%) in Standard Care arm, p = 0.63. Fig. 4B shows Kaplan Meier plot for freedom from prediabetes or diabetes, which also was not different in both groups.

Arterial hypertension was diagnosed before transplantation in almost all transplant recipients (Table 1) and measured blood pressure values were not different for Slow & Low arm (systolic: 136.4 ± 1.4, diastolic: 80.0 ± 0.9) versus Standard Care arm (systolic: 135.2 ± 1.4, diastolic: 79.3 ± 0.9) at 6 months visit (Table 2). Also all values for cholesterol, HDL, LDL, weight or BMI were similar in both arms (see Table 2). The per-protocol analysis revealed similar results in all endpoints.

#### Subgroup analyses

**Fig. 5:**
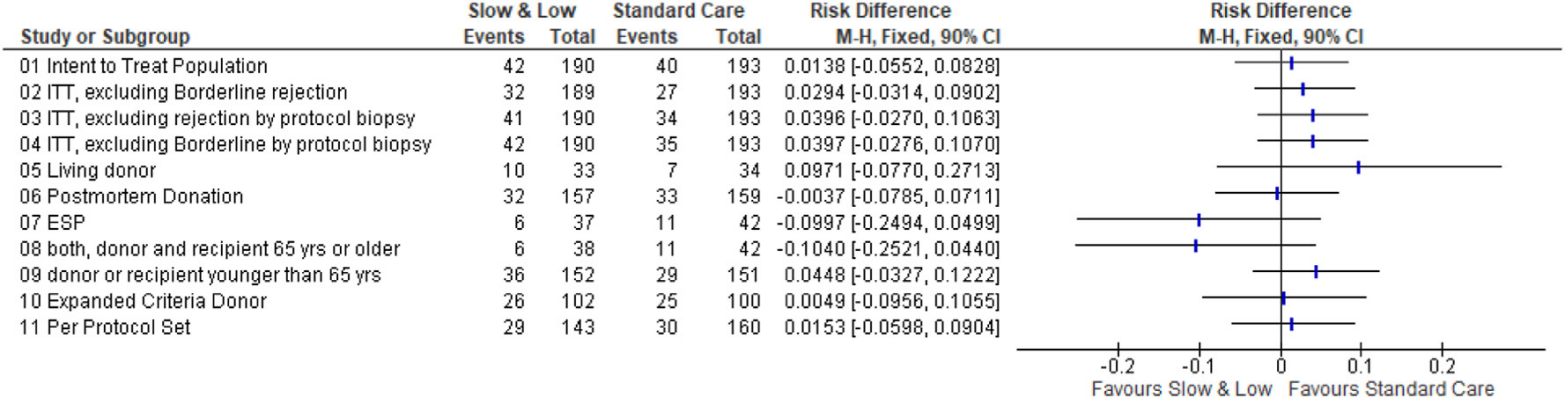
Forest plot of risk differences in different populations (intent to treat, per protocol set), in various sensitivity analyses (counting of events: excluding borderline rejections or rejections detected in protocol biopsies), or subgroups. The non-inferiority criterion defined for intent-to-treat population is not met if the upper limit of the 90 percent two-sided confidence intervall is higher than 0.1250.

While statistical analysis of study subgroups is complicated due to low case and event numbers, it nevertheless may be important for the right interpretation of the study results and its major determinants (Fig. 5). Besides different ITT evaluations (including/excluding borderline rejections/protocol biopsies), also per-protocol set of patients confirmed non-inferiority of Slow & Low arm compared to Standard Care arm. In addition, our data suggest that the Slow & Low arm is non-inferior in the large group of postmortem donor transplantation as well as in donors with ECD criteria, but less convincing in the small group of living donations. Hereby, apparently recipient age seems to play a major role, since the group of the ESP study participants or the recipient group older than 65 years even showed a tendency towards superiority for Slow & Low arm (Fig. 5). In contrast, primary end point results were intermediate in donor or recipients younger than 65 years.

## Discussion

This investigator-initiated, open-label, randomized, multicenter study was performed in an immunological low-risk patient population to compare efficacy of a fixed dose, bottom-up, slowly incremental tacrolimus saturation approach up to reduced trough levels (S&L study) compared to the current gold standard protocol of immunosuppressive therapy after renal transplantation.^1^ The approach as performed in this study markedly simplified the tacrolimus drug saturation process, since trough levels during the first six days were blinded to the investigators and no single dose correction was done before day 7 after renal transplantation. For the event of reaching a potentially toxic tacrolimus drug level of 20 μg/ml or higher on two days, a specific alarm system was implemented to unblind and inform the investigators for correction of the fixed 5 mg dose of tacrolimus. This seemed necessary considering large intra- and interindividual pharmacokinetic differences regarding tacrolimus blood levels. These depend on metabolisation rate and bioavailability, as well as patient factors such as age, sex, weight, CYP3A5 expression level, and food and drug interactions.^15^^,^^16^ Higher peak and lower trough levels are found in rapid metabolisers, while slow metabolisers more often achieve tacrolimus blood levels outside the therapeutic window. Due to these large pharmacokinetic variabilities, individualised dosing with regular therapeutic monitoring from the beginning has always been proposed.^17^ Nevertheless, since only one patient had to be intervened for adaption of the fixed dose approach, omitting the alarm system seems reasonable for implementation of the Slow & Low concept in the future.

Most importantly, the primary study aim demonstrating non-inferiority regarding the combined endpoint of BPAR, graft loss, and death was reached by the fixed dose tacrolimus Slow & Low arm compared to the Standard Care arm. This overall result was robust and independent of the inclusion or exclusion of borderline or protocol rejections in the primary endpoint. This is the first study to show that therapeutic drug monitoring for prolonged release tacrolimus using an initially fixed dose of 5 mg can be abandoned until start of the second week after renal transplantation, when the relationship of tacrolimus dose to drug level stabilizes due to ceasing corticosteroid interference and likely post-surgery bowel movement normalization.^7^ The overall result of non-inferiority of the low fixed dose tacrolimus arm compared to the current standard immunosuppressive regimen demonstrates this novel approach efficient and safe and may be a cost effective alternative due to its simplified handling of patients early after transplantation.

For the interpretation of this study result, it has to be considered that tacrolimus trough levels were severely different in both groups during the first month after transplantation, remained different further on but approached each other progressively with time after transplantation. Apparently, investigators in transplant studies are very conservative and reluctant to lower drug levels for fear of consequences of rejection. More than a decade after publication of the ELITE Symphony trial, this described phenomenon has been seen in many studies and apparently need to be considered for interpretation of the results of this study as well.^1^ While in the Standard Care treatment arm mean tacrolimus trough levels always remained at the high end of the target, in the low fixed dose arm mean tacrolimus levels were frequently even outside the higher target range at later time points.

Besides no difference in the overall percentage of BPAR, a slight but significant shift of BPAR towards more severe acute T-cell mediated rejections (mainly from borderline to BANFF IA in post-hoc analysis) in Slow & Low arm could be a very sensitive indicator of the reduced immunosuppressive exposure compared to the Standard Care control arm. At the same time, the frequency of humoral rejections and the *de novo* appearance of total or donor specific anti-HLA antibodies were not affected. While this apparently had no severe consequences in regard to other safety parameters, long-term follow-up may be important and demonstrates a relatively sensitive relationship of very early tacrolimus trough levels in regard to the severity (post-hoc analysis) but not frequency (pre-specified analysis) of T-cell-mediated rejections within the six months study period. As done in our study, recent studies by others support the inclusion of borderline rejections in the definition of BPAR (even when found in protocol biopsies).^18^ They show higher rates of subsequent rejection, progressive nephron loss, impaired renal graft function, and higher rates of donor-specific antibodies and graft failure in the clinical course after borderline rejections. Nevertheless, reduced early tacrolimus exposure led to a marked predominance of all primary events in the Slow & Low arm (84%) within the first month after transplantation compared to only 43% in the Standard Care arm, where the events were more equally distributed over the full six months observation period (exploratory analysis). Although we do not know the general consequence/relevance of the BPAR event timing yet, the event predominance early on could be an advantage of the fixed low dose in the handling of the transplant recipient, when very frequent visits and a short reaction time is given. Nevertheless, this may clearly dependent on country and center specific patient handling strategies.

The fixed, slower and lower tacrolimus exposure was also meant to potentially improve toxicity associated with the use of this calcineurin inhibitor, but no clear cut benefit for any secondary outcome parameter such as AE, SAE, kidney function, neurotoxicity (some tendency), or the incidence of DGF was found in the overall population. Even the high tacrolimus levels during the first week in the Standard Care control arm compared to really low levels in the Slow & Low arm did not lead to a significant increase in the rate of DGF suggesting no severe role of acute CNI toxicity in mediating DGF in this setting. Interestingly, even at the earlier time points when tacrolimus trough levels were still quite different, not even a tendency for improved kidney function was seen in the low exposure arm. In addition, the initially clearly lower tacrolimus exposure did not demonstrate any advantageous effect in relation to the incidence of important cardiovascular risk factors such as PTDM, arterial hypertension, or hyperlipidemia during study period. This is in contrast to our experience using a rapid steroid withdrawal approach when tacrolimus/MMF based immunosuppression combined with induction therapy was able to almost bisect the incidence of PTDM during the first year after renal transplantation.^6^ Whether or not a longer and more severe reduction of tacrolimus trough levels has to be done to decrease the combined diabetogenic toxicity of tacrolimus/corticosteroids remains speculative. Similarly, no effect on the incidence of general or CMV infections, but some tendency towards a reduced incidence regarding BKV infection/nephropathy was noted. Five malignancies were detected during the six months follow-up period, all of which occurred in the Slow & Low arm. Since immunosuppressive drug exposure was lower in the Slow & Low arm compared to the Standard Care arm, this cancer distribution most likely occurred by chance.

Several limitations of our study need to be considered. The investigators were quite reluctant to keep the low tacrolimus trough levels of the Slow & Low arm within the relatively narrow and low margins at later time points (≥2 months) of the study, whereas the fixed dose saturation worked exceptionally well for early drug target range in the first week. We cannot completely exclude that the lack of blinding influenced the study outcome, since the decision for an indication biopsy involves subjective interpretation. Nevertheless, the biopsy frequency was numerically even higher in the Slow & Low arm increasing the chance for detection of BPAR. Another weakness of the study is that the follow-up period of six months is quite short to examine an influence on the development of donor-specific antibodies with impact on the long-term transplant outcome. A six months follow-up is also to short to already demonstrate a lasting effect on the incidence of cancer, cardiovascular events or mortality. Clearly, these study results are limited to an immunologically low-risk population of Caucasian ethnicity and cannot be extrapolated to a different study population with other ethnicities or an immunologically high-risk constellation. It remains to be mentioned that no patients with T-lymphocyte depleting induction therapy are included in the study.

A strength of the study is the fact that by reflecting the typical renal transplant situation in Germany/Europe relatively old donors and severly diseased elderly transplant recipients were included, which seem to be especially suited for such a low exposure protocol. The ECD criteria donor has become the standard donor by now as its percentage in this study as well as in Germany is at 55%. Despite these complicated baseline conditions, excellent results six months after renal transplantation were reached considering high patient and graft survival, and with 92% a high rate of patients finishing the full study period.

With the limitation of much lower case numbers for all subgroup analyses, these still remain important for the generation of further hypotheses as well as for the right interpretation of the study results and its major determinants. Hereby, it is remarkable that the Slow & Low approach regarding its primary combined endpoint appears to be broadly applicable for any deceased or ECD donor. While living donor numbers were low and applicability according to the results unclear, it may be important that the average recipient age of living donation was 47 ± 13 and for deceased donation was 56 ± 12 years. According to our study results, the Slow & Low approach may be especially applicable for renal transplantation within the ESP program and in general for recipients at least 65 years old.

In conclusion, our large investigator-initiated trial compared the gold standard immunosuppressive regimen with a bottom-up, slowly incremental, fixed dose tacrolimus saturation approach. For the first time in renal transplantation, therapeutic drug monitoring was abandoned within the first week. Reduced tacrolimus trough levels were targeted later on, although these were not consistently achieved in the Slow & Low arm during follow-up period. This simplified drug approach was non inferior in efficacy and safety parameters in a broad range of transplant recipients and may be considered to replace the current standard drug monitoring approach accompanied by frequent dose changes.

## Contributors

CH and MO contributed to study design. CH and JS contributed to data collection, data interpretation and writing of the manuscript. CH and JS also accessed and verified the data. All authors were involved in the provision of study materials and patients and provided critical revision with important intellectual content, especially KB. All authors had full access to all the data in the study, and had final responsibility for the decision to submit for publication. All authors have approved the final version for submission.

## Data sharing statement

The Coordination Centre for Clinical Trials, Faculty of Medicine Carl Gustav Carus, Dresden University of Technology, Dresden, Germany, as an independent contract research organization was responsible for regulatory affairs, data management, monitoring and statistical analyses.

## Declaration of interests

CH received grants from Astellas Pharma GmbH during the conduct of the study as well as grants and other from Novartis, other from Astellas, other from Chiesi, grants and other from AstraZeneca, grants and other from Hansa Biopharma, other from Alexion, grants and other from Boehringer Ingelheim, other from Takeda, other from Vifor Pharma, grants and other from Otsuka, other from Stadapharm, other from GlaxoSmithKline, other from Bayer Vital, other from MSD, grants from Calliditas, grants from Vera Therapeutics outside the submitted work.

OW has received research grants for clinical studies, speaker's fees, honoraria and travel expenses from Amgen, Alexion, Astellas, Basilea, Biotest, Bristol-Myers Squibb, Correvio, Chiesi, Gilead, Hexal, Janssen, Dr. F. Köhler Chemie, MSD, Novartis, Roche, Pfizer, Sanofi, TEVA, AstraZeneca, GSK and UCB.

KB received grants from Astellas during the conduct of the study; grants from Astellas, Chiesi, Alexion, CSL-Behring, MSD, personal fees from MSD, Takeda, Natera, Aicuris, CareDx, Stada, CSL-Behring, Paladin, Veloxis, Alexion, Pirche, Vifor, Carealytics, Neovii, outside the submitted work.

TV received personal fees from Astellas Pharma GmbH outside the submitted work.

ET received grants from University Hospital Carl Gustav Carus at the Technische Universität Dresden, Department of Internal Medicine III, Division of Nephrology during the conduct of the study.

All other authors of this manuscript have no conflicts of interest to be disclosed.

## Appendix A Supplementary data

Supplementary Material clean version

Supplementary Material Additional Investigators
