# Supplementary material for: Fixed low dose versus concentration-controlled initial tacrolimus dosing with reduced target levels in the course after kidney transplantation: results from a prospective randomized controlled non-inferiority trial (Slow & Low study)
Source: eClinicalMedicine. 2023 Dec 22;67:102381. doi: 10.1016/j.eclinm.2023.102381 (PMC10751828; doi:10.1016/j.eclinm.2023.102381)
Supplement: Supplementary Material clean version [file mmc1.docx]

# Supplementary Material

**Table of Contents -** [**Supplementary Material** 1](#_Toc129078022)

[Supplementary methods: 1](#_Toc129078023)

[Inclusion and Exclusion Criteria 1](#_Toc129078024)

Further secondary end points 2

Prophylaxis against infection 2

[Severe Protocol Violations 3](#_Toc129078025)

Supplementary Results: 4

Induction and maintenance therapies 4

[Supplementary Figure: Study Design 5](#_Toc129078026)

## Supplementary methods

## Inclusion and Exclusion Criteria

Inclusion Criteria

1. Male or female allograft recipients at least 18 years old

2. Primary or secondary kidney transplantation

3. Deceased or living dondor

4. Normal immunological risk profile,

- PRA level <= 20 %,
- AB0-compatible donation,
- negative crossmatch

5. Written informed consent of the patient

6. Allowance to contact the families doctor and/or neprologist for study relevant data

Exclusion Criteria

1. Former graft loss due to severe rejection within the first year after transplantation (in case of secondary transplantation)

2. Multiorgan recipient

3. Patients receiving a kidney from a „non heart beating“ donor

4. Complete HLA-identical living donor (twins)

5. Patients with a history of malignancy during the last five years (except squamous or basal cell carcinoma of the skin after successful treatment)

6. Patients with uncontrolled infectious disease, particularly patients who are HIV-positive or suffer from chronic hepatitis B or C or tuberculosis

7. Patients with severe gastroenteric disorder, particularly severe diarrhoe and symptoms of enteric malabsorption

8. Patients suffering from liver cirrhosis Child B or C or other severe liver disease (ASAT, ALAT, GammaGT ≥ 3-fold increased)

9. Thrombopenia <70·000/mm^3^

10. Leukopenia <2·500/mm^3^

11. Participation in another clinical trial within the last 4 weeks prior to inclusion

12. Addiction or other disorders that do not allow the person concerned, to estimate the nature, scope and possible consequences of the clinical trial

13. Pregnant or breast-feeding women

14. Women of childbearing age, except women who meet any of the following criteria:

- post-menopausal (12 months natural amenorrhea or 6 months amenorrhea with serum > 40 U / ml)
- postoperatively (6 weeks after bilateral ovarectomy with or without hysterectomy)
- regular and correct use of a contraceptive method with error rate < 1 % per year (e· g· implants, depot injections, oral contraceptives, intrauterine device)
- sexual abstinence
- vasectomy of the partner

15. Evidence that the patient is likely to fail to comply with the protocol (e· g· lack of cooperation)

16. Hypersensitivity to tacrolimus

**Further secondary end points**

Two different definitions for DGF were considered: DGF was defined as the need for dialysis after transplantation, i.e. at least one dialysis within 4 days after TX followed by at least one other dialysis until day 7. The need for dialysis must not be caused by graft rejection or graft loss. Alternatively, DGF was defined by center standard. Clinical assessment parameters included vital signs and laboratory analyses designed to determine the incidence of all adverse and serious adverse events, infections, malignancies and death throughout the study.

**Prophylaxis against infection**

All patients except recipient and donor cytomegalovirus (CMV seronegative) received at least a trimonthly prophylaxis with valganciclovir according to guidelines. Additionally, Pneumocystis jirovecii pneumonia (PJP) prophylaxis with trimethoprim/sulfamethoxazole or pentamidine was mandatory for all patients for at least 6 months.

## Severe Protocol Violations

The following severe protocol deviations led to a patient’s exclusion from the per-protocol set:

1. violation of inclusion or exclusion criteria
2. Advagraf discontinued and not followed by any other treatment with tacrolimus (Prograf, generic medicinal product)
3. induction therapy with basiliximab:
   - less than two doses
   - two doses not applied between day -1 and 14 (day 0 = day of transplantation)
4. steroids discontinued within 5 months after transplantation
5. administration of MMF/CellCept
   - interrupted for consecutive 22 days or longer
   - dose reduced below 2 x 500 mg CellCept or 2 x 360 mg Myfortic for 22 consecutive days or longer
6. additional immunosuppression to the 3-fold combination predefined in the protocol

Any exception regarding immunosuppressive treatment defined in the study protocol were taken into consideration.

## Supplementary Results

**Induction and maintenance therapies**

The first dose of 20 mg basiliximab was given pre- or intraoperatively in 99·2% (395/398) and the second dose was administered on postoperative day 4 in 98·2% (391/398) of the randomized patients.

As already reported in the manuscript, target trough levels for tacrolimus were within the predefined therapeutic window in Standard Care arm but only during the first month in the Slow&Low arm (see Figure 2), thereafter tacrolimus trough levels became more overlapping with the Standard Care arm. One patient had to be intervened for adaption of the fixed dose approach as predefined by tacrolimus trough levels twice > 20 µg/ml during that period. The accompanying immunosuppressive drug MMF/MPA was administered with an average daily dose of 1827·8 ± 264·2 mg in Standard Care arm and 1783·6 ± 295·6 mg in Slow&Low arm, without any differences between study arms.

## Supplementary Figure: Study Design


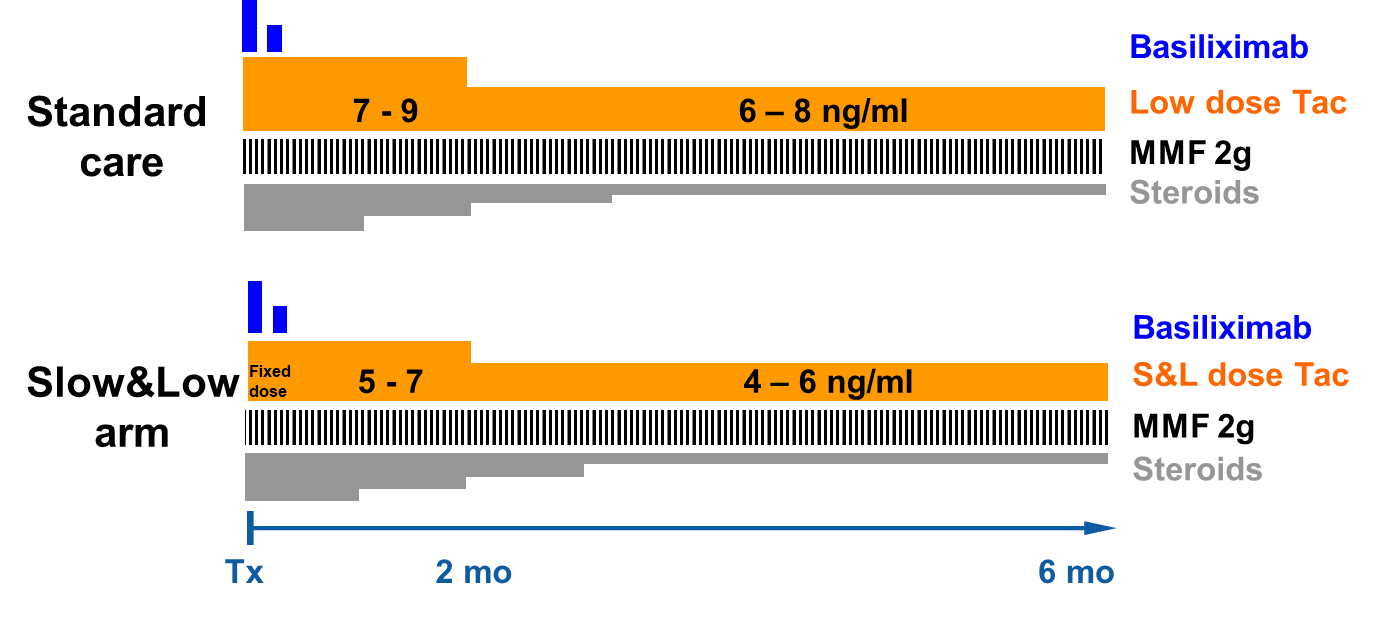


Supplementary Figure: The study design over time is presented. All patients received induction therapy with basiliximab (Simulect®, Novartis, 20 mg intravenously on day 0 before allograft reperfusion and day 4), prolonged release tacrolimus administered once daily (Advagraf®, Astellas Pharma GmbH; represented by bands where a thinner orange band corresponds to a lower target through level), 2 x 1 g/die mycophenolate mofetil (MMF) (CellCept®, Roche Pharma AG) or enteric coated mycophenolic sodium (EC-MPS) at equivalent doses (black and white striped band), and prednisolone (Solu Decortin®, Merck Serono), all of which were started before transplantation. Prednisolone/Methylprednisolone was given at center standard, but tapering (shown as gray bands where a thinner gray band corresponds to a lower dose) had to be done to reach 20 mg/16 mg after four weeks, 10 mg/8 mg after eight weeks, and 5 mg/4 mg daily after 12 weeks, respectively. In Standard Care arm , tacrolimus starting dose was 0·2 mg/kg once daily followed by tacrolimus trough levels of 7-9 ng/ml in the first two months and 6-8 ng/ml from month 3 to 6. Trough levels until day 6 had to be determined on a daily basis and later according to center standard. In the Slow&Low arm, prolonged release tacrolimus was started once daily at a fixed dose of 5mg before and for the first six days after transplantation (as printed in panel B). Trough levels until day 6 had to be determined on a daily basis but were blinded to the investigators. The first unblinded tacrolimus trough level on day 6 led to the first dose correction on day 7 by the investigators. From day 7 on, tacrolimus trough levels were adapted to reduced levels of 5-7 ng/ml in the first two months and 4-6 ng/ml from month 3 to 6. S&L = Slow & Low study.
