# Supplementary material for: Fixed low dose versus concentration-controlled initial tacrolimus dosing with reduced target levels in the course after kidney transplantation: results from a prospective randomized controlled non-inferiority trial (Slow & Low study)
Source: eClinicalMedicine. 2023 Dec 22;67:102381. doi: 10.1016/j.eclinm.2023.102381 (PMC10751828; doi:10.1016/j.eclinm.2023.102381)
Supplement: Supplementary Material Additional Investigators [file mmc2.docx]

**Appendix**

Additional investigators who participated in the German S&L STUDY are as follows:

Prof. Dr. med. habil. Matthias Girndt (Universitätsklinikum Halle/Saale), Prof. Dr. Gunter Wolf (Universitätsklinikum Jena), Prof. Dr. Christine Kurschat (Universitätsklinikum Köln), PD Dr. Kai Lopau (Universitätsklinikum Würzburg), Univ.-Prof. Dr. Jens Lutz (Universitätsklinikum Mainz)
